# Response to Letter to the Editor Entitled “Unsafe Care and Fake News in Freeman‐Burian Syndrome”

**DOI:** 10.1002/ccr3.70079

**Published:** 2025-01-06

**Authors:** Aiji Sato

**Affiliations:** ^1^ Department of Anesthesiology Aichi Gakuin University School of Dentistry Nagoya Japan

## Author Contributions

**Aiji Sato:** formal analysis, writing – original draft.

Dear Authors of the LTE, Editor‐in‐Chief, CCR Readers,

We appreciate your thoughtful feedback regarding our case report on facioscapulohumeral muscular dystrophy (FSS). We fully understand the importance of accuracy in medical literature and appreciate the concern regarding the spread of potentially inaccurate information.

As we emphasized in our previous response, we followed best practices in the treatment of a patient who had already been diagnosed with FSS by another physician, and we stand by the validity of the case as presented. We fully understand the importance of selecting appropriate and accurate references when preparing a case report. Our intention was to rely on available literature to support our report, aiming to provide a well‐rounded perspective. If our paper contains inaccurate or outdated references, we would appreciate constructive suggestions on how to address any potential issues. We understand the concern about avoiding misleading papers, and receiving recommendations for incorporating more appropriate sources would be greatly helpful.

Additionally, while it is true that our report is based on a single case, as mentioned in your letter, this is often inevitable when dealing with rare conditions such as FSS. In such situations, it is not common to rely on individual case studies to contribute to the broader medical literature. We believe our report was presented in good faith, following best practices, even while working within the inherent limitations of rare case studies.

We did have photographs of the distinctive facial features associated with the syndrome, but we chose not to include them out of respect for the patient's wishes. We fully understand the significant value such images can offer in understanding rare cases, but patient consent and privacy must remain a priority.

In terms of sedation, we meticulously managed the process, ensuring that no respiratory complications occurred post‐anesthesia. Our advanced medical facility and multidisciplinary approach, which involved close collaboration with a respiratory specialist, allowed us to tailor the sedation plan to the patient's specific needs. The respiratory specialist determined that intubation was unnecessary, thus reducing potential risks. This careful coordination reflects our commitment to patient safety and best practices, particularly when managing complex cases like this.

Our pain management approach was similarly successful, avoiding any respiratory depression while providing adequate pain relief. By selecting appropriate analgesics and closely monitoring the patient, we maintained stable respiratory function and prevented any compromise to the patient's airway or breathing. There were no safety issues throughout the treatment, and our strategy was guided by a deep understanding of the patient's medical condition and a commitment to safety and comfort at all times.

At the time, we believed that we had chosen the safest and most effective method for managing the patient's condition. Based on the available clinical information, we carefully weighed the risks and chose an approach that provided the best balance between effective pain management and patient safety. While we are always open to reflection and improvement, our actions were guided by a genuine commitment to delivering the safest and most effective care possible.

We fully recognized the patient's decreased respiratory function, which was a significant factor in our treatment planning. This heightened the need for meticulous monitoring and a tailored approach to both sedation and pain management. Through close collaboration with specialists and continuous evaluation of the patient's respiratory status, we managed these complexities while prioritizing safety throughout the treatment process.

We believed that managing the patient without intubation would be the best approach, as avoiding invasive airway management could reduce complications and improve the overall recovery experience. We made every effort to implement what we considered the safest and most appropriate plan for the patient's specific needs. Throughout the process, we continually assessed the patient's condition to ensure that our approach remained effective and safe, maintaining our commitment to the highest standard of care.

Looking ahead, we are committed to applying the lessons learned from this case to improve our handling of similarly rare and complex cases. Every case presents unique challenges, and this experience has deepened our understanding of how to approach such conditions with greater confidence and precision. The insights we have gained—from patient management strategies to the importance of interdisciplinary collaboration—will serve as a foundation for enhancing our practices. Moving forward, we will utilize these lessons to improve patient outcomes, prioritize safety, and refine our approach to rare medical scenarios, while continuing to provide individualized, evidence‐based care.

We sincerely appreciate your feedback and hope that this letter clarifies our perspective.

